# Beyond GH stimulation tests: genetic heterogeneity and treatment response in children with diagnosed GH deficiency

**DOI:** 10.1210/jendso/bvag106

**Published:** 2026-05-07

**Authors:** Lukas Plachy, Petra Dusatkova, Lukas Kavciak, Shenali Anne Amaratunga, Matvei Slavenko, Jana Drabova, Klara Maratova, Vit Neuman, Barbora Obermannova, Stanislava Kolouskova, Marta Snajderova, Zdenek Sumnik, Jan Lebl, Stepanka Pruhova

**Affiliations:** Department of Pediatrics, Motol and Homolka University Hospital, Prague 15000, Czech Republic; Department of Pediatrics, Second Faculty of Medicine, Charles University, Prague 15000, Czech Republic; Department of Pediatrics, Motol and Homolka University Hospital, Prague 15000, Czech Republic; Department of Pediatrics, Second Faculty of Medicine, Charles University, Prague 15000, Czech Republic; Department of Pediatrics, Motol and Homolka University Hospital, Prague 15000, Czech Republic; Department of Pediatrics, Second Faculty of Medicine, Charles University, Prague 15000, Czech Republic; Department of Pediatrics, Motol and Homolka University Hospital, Prague 15000, Czech Republic; Department of Pediatrics, Second Faculty of Medicine, Charles University, Prague 15000, Czech Republic; Department of Pediatrics, Motol and Homolka University Hospital, Prague 15000, Czech Republic; Department of Pediatrics, Second Faculty of Medicine, Charles University, Prague 15000, Czech Republic; Institute of Biology and Medical Genetics, Motol and Homolka University Hospital, Prague 15000, Czech Republic; Department of Pediatrics, Motol and Homolka University Hospital, Prague 15000, Czech Republic; Department of Pediatrics, Second Faculty of Medicine, Charles University, Prague 15000, Czech Republic; Department of Pediatrics, Motol and Homolka University Hospital, Prague 15000, Czech Republic; Department of Pediatrics, Second Faculty of Medicine, Charles University, Prague 15000, Czech Republic; Department of Pediatrics, Motol and Homolka University Hospital, Prague 15000, Czech Republic; Department of Pediatrics, Second Faculty of Medicine, Charles University, Prague 15000, Czech Republic; Department of Pediatrics, Motol and Homolka University Hospital, Prague 15000, Czech Republic; Department of Pediatrics, Second Faculty of Medicine, Charles University, Prague 15000, Czech Republic; Department of Pediatrics, Motol and Homolka University Hospital, Prague 15000, Czech Republic; Department of Pediatrics, Second Faculty of Medicine, Charles University, Prague 15000, Czech Republic; Department of Pediatrics, Motol and Homolka University Hospital, Prague 15000, Czech Republic; Department of Pediatrics, Second Faculty of Medicine, Charles University, Prague 15000, Czech Republic; Department of Pediatrics, Motol and Homolka University Hospital, Prague 15000, Czech Republic; Department of Pediatrics, Second Faculty of Medicine, Charles University, Prague 15000, Czech Republic; Department of Pediatrics, Motol and Homolka University Hospital, Prague 15000, Czech Republic; Department of Pediatrics, Second Faculty of Medicine, Charles University, Prague 15000, Czech Republic

**Keywords:** growth hormone deficiency, short stature, next-generation sequencing, RASopathies, growth plate, growth hormone treatment

## Abstract

**Context:**

Growth hormone (GH) stimulation tests have limited reliability and may lead to false-positive results.

**Objective:**

This work aimed to evaluate genetic causes of short stature in children clinically diagnosed with GH deficiency (GHD) and assess genotype-phenotype correlations.

**Methods:**

A retrospective single-center study was conducted including 233 children with clinically diagnosed primary GHD treated with GH for 5 years or more. Genetic testing was performed using a targeted next-generation sequencing panel of 398 growth-associated genes.

**Results:**

A genetic cause was identified in 39 of 233 (17%) children. Only 13 of 39 (33%) findings confirmed GHD (genes *CHD7*, *GH1, GHSR*, *GLI2*, *GNAO1*, *KMT2D*, *OTX2* [3], *POU1F1*, *PROP1*, *SALL4*, *TBX3*). The remaining 26 of 39 (67%) revealed alternative etiologies of growth failure: RASopathies (13/39; genes *NF1* [2], *PTPN11* [7], *RAF1*, *SOS1* [2], *SPRED1*), growth plate disorders (8/39; genes *ACAN*, *COL2A1*, *EXT2*, *FGFR3* [2], *NPR2* [3]), and miscellaneous conditions (5/39; genes *CDC42*, *LMNA*, *HMGA2*, *PMM2*, *RAI1*). Genetically confirmed GHD patients presented with lower peak GH (median 1.8 vs 4.8 [alternative genetic etiology] and 5.9 μg/L [no genetic etiology]; *P* = .017), more frequent combined pituitary hormone deficiency (46% vs 4% and 12%; *P* = .001) and magnetic resonance imaging midbrain abnormalities (73% vs 14% vs 32%; *P* = .003). Conversely, the alternative etiology group demonstrated the least robust 5-year GH therapy response (median height gain 1.4 vs 2.3 [GHD group] and 1.8 [no genetic etiology]; *P* = .001).

**Conclusion:**

The population of children clinically diagnosed with GHD is genetically heterogeneous. A statistically significant proportion harbors defects in RAS-MAPK signaling or growth plate disorders, highlighting the limitations of current methods of GHD diagnostics.

The diagnosis of growth hormone deficiency (GHD) relies on a combination of auxological assessment, biochemical testing, and radiological imaging. In children with suspected GHD, GH stimulation tests are performed to assess the secretory capacity of the pituitary gland. Currently, these tests remain the gold standard for confirming the diagnosis [1, 2].

However, a major limitation of this diagnostic approach lies in the limited reliability of GH stimulation testing [3, 4]. The results are highly variable, influenced by numerous physiological and technical factors [5-8], and may yield false-positive results [9]. Consequently, concerns have been raised regarding the validity of current GHD diagnostic criteria [4]. It has been proposed that children clinically diagnosed with GHD represent an etiologically heterogeneous group with diverse underlying causes of growth failure, many of which are unrelated to GH secretion or function [10]. This presumption lacks comprehensive evaluation in clinical studies. Although genetic approaches have demonstrated strong potential in clarifying the causes of short stature [11-16], studies focused on GHD cohorts have largely targeted genes involved in GH production or secretion [17-20], potentially overlooking other etiologies of impaired growth.

The aim of this study was to comprehensively evaluate the genetic etiology of short stature in children clinically diagnosed with GHD and to assess correlations between genetic findings and clinical phenotypes including response to GH treatment.

## Materials and methods

### Study population

Children with a clinical diagnosis of primary GHD were retrospectively identified from the institutional database of children treated with GH at the Centre of Pediatric Endocrinology of Motol University Hospital, Prague, Czech Republic (database search performed in January 2024). Children were enrolled if they met the following criteria: 1) confirmed diagnosis of GHD (details discussed later); 2) absence of secondary causes of GHD (eg, brain tumor, neurosurgical intervention, cranial irradiation); 3) continuous GH treatment at our center for a minimum of 5 years; and 4) written informed consent for participation in the study (including genetic testing) provided by parents or legal guardians. The study protocol was approved by the institutional ethics committee of the Motol University Hospital and the Second Faculty of Medicine, Charles University, Prague (No. EK-753.3.5.21).

### Clinical evaluation

Data regarding birth parameters, medical history, previous height development, GH treatment, and results of laboratory and radiological examinations were obtained from the medical records. Parental height was measured using a wall-mounted stadiometer to the nearest 1 mm. All anthropometric data were standardized using recent normative values [21, 22]. The clinical diagnosis of GHD was established in accordance with current international guidelines [1, 2]. GH stimulation testing was performed in children presenting with auxological and/or clinical features suggestive of GHD (height < −3 SD; and/or height < −2 SD with a decrease of height SD score [SDS] of >0.5 over 1 year in children age >2 years; and/or height <1.5 SD below midparent height; and/or height velocity < −2 SD sustained over 1 year or <−1.5 SD sustained over 2 years; and/or other pituitary hormone deficiency) [2] and a serum insulin-like growth factor type 1 (IGF-1) concentration below 0 SD for age and sex. Serum IGF-1 and GH concentrations were measured using automated immunoassays (Immunodiagnostic Systems; IGF-1: catalog No. IS-3900, RRID:AB_2861357; GH: catalog No. IS-3700, RRID:AB_2861356). GHD was confirmed in children with a peak GH concentration less than 10 μg/L in both clonidine and insulin tolerance tests. Sex-steroid priming (estradiol 2 mg administered 2 evenings preceding the test) was administered to all prepubertal children aged 9 years or older prior to stimulation testing.

### Genetic testing

Genomic DNA was extracted from peripheral blood (QIAamp Blood Mini Kit, Qiagen). All girls had karyotype examination to exclude Turner syndrome. Children with a clinical phenotype suggestive of a specific genetic disorder underwent targeted genetic testing via Sanger sequencing or array comparative genomic hybridization. In children without identified causal variant in first-tier testing, or in cases in which no specific genetic disorder was suspected based on phenotype, next-generation sequencing (NGS) was performed using a custom-designed targeted panel comprising 398 growth-associated genes (for information about specific families, see supplementary data provided in the online repository [23]). All variants identified by NGS were confirmed by Sanger sequencing, as previously described [24]. Variant filtering was based primarily on low or absent frequency in control databases (eg, GnomAD, ExAc, and 1000 genomes). Functional impact was predicted using multiple in silico tools, including REVEL, CADD, DANN, SIFT, PolyPhen2, MutationTaster, and SpliceAI. The localization of variants within functionally relevant protein domains was also considered. Copy number variants were analyzed from NGS data using DECon software [25].

All variants with potential clinical importance were evaluated by American College of Medical Genetics and Genomics standards and guidelines [26]. Variant interpretation was supported by Franklin software (https://franklin.genoox.com version date May 3, 2024), which incorporates American College of Medical Genetics and Genomics criteria with rule strength (very strong, strong, moderate, or supporting). When applicable, rule strength was modified based on extended database investigation and detailed clinical evaluation. Familial segregation analysis was performed obtaining DNA samples and height data from available family members. The guidelines formulated by Jarvik and Browning [27] were followed to incorporate cosegregation data in the pathogenicity classification. In the end, all genetic variants were classified as pathogenic (P), likely pathogenic (LP), benign (B), likely benign (LB), or as variants of uncertain significance (VUS).

### Statistical evaluation

Based on the analysis of the genes in which causal variants were found, children were stratified into 3 groups: those with genetic variants causing GHD (GHD group), variants accounting for short stature due to an alternative genetic etiology (alternative etiology group), and children with no causal genetic variant identified (no genetic etiology group). In addition, the children were stratified into 4 groups based on their peak stimulated GH concentration (<3.0 μg/L, 3.0-4.9 μg/L, 5.0-6.9 μg/L, and 7.0-9.9 μg/L).

Clinical parameters were compared among groups that were formed based on genetic examination and peak stimulated GH concentrations. Comparisons among the groups were performed using the Kruskal-Walis test for continuous variables, and categorical variables were compared using the Pearson chi-square test. To identify differences between specific pairs of groups, post hoc analyses were conducted. For continuous variables, Dunn's test with Bonferroni correction for multiple comparisons was applied. For categorical variables, pairwise comparisons were performed using the Fisher exact test with Bonferroni correction. *P* values less than .05 were considered statistically significant.

## Results

Of the 259 children diagnosed with primary GHD who received GH therapy at our center for a minimum duration of 5 years, parents/legal guardians of 233 (72 girls) provided informed consent for genetic testing, and these children were subsequently enrolled in the study. The characteristics of the study cohort are summarized in Table 1.

**Table 1:** Characteristics of the study cohort

Birth weight adjusted for gestational age (SD)	−0.8 (−1.6 to −0.2)
Birth length adjusted for gestational age (SD)	−1.5 (−2.1 to −0.9)
Neonatal jaundice with phototherapy (n; %)	43 (18%)
Neonatal hypoglycemia (n; %)	26 (11%)
Cryptorchidism (n; % of boys)	22 (14%)
Age at GH therapy initiation, y	4.7 (3.4 to 6.5)
Height at GH therapy initiation (SD)	−3.0 (−3.3 to −2.6)
Pretreatment IGF-1 below detection limit 15 μg/L (n; %)	25 (11%)
Pretreatment IGF-1*^a^* (SD)	−1.7 (−1.9 to −1.3)
Peak stimulated GH, μg/L	5.7 (3.9 to 7.4)
Combined pituitary hormone deficiency (n; %)	30 (13%)
Central adrenal insufficiency (n; %)	27 (12%)
Central hypothyroidism (n; %)	25 (11%)
Hypogonadotropic hypogonadism (n; %)	6 (3%)
AVP deficiency (n; %)	2 (1%)
MRI performed (n; %)	214 (92%)
MRI midline abnormality (n; %*^b^*)	69 (30%)

Results are expressed as medians and interquartile ranges or as number and percentage of children.

Abbreviations: AVP, arginine vasopressin; GH, growth hormone; IGF-1, insulin-like growth factor type 1; MRI, magnetic resonance imaging; n, number of children.

^*a*^Only children with detectable pretreatment IGF-1 concentrations were included in the analysis.

^*b*^Percentage of children with MRI performed.

**Figure 1:**
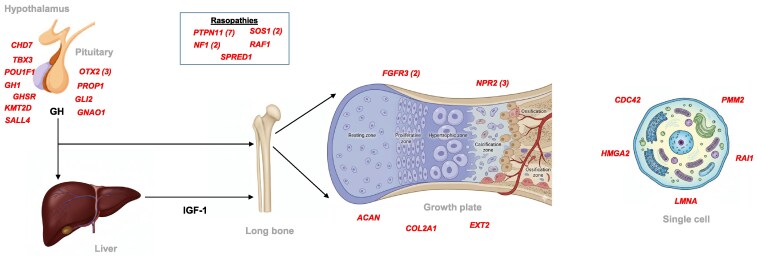
Genetic etiology of growth disorders in children clinically diagnosed with growth hormone deficiency.

A genetic cause of short stature was identified in 39 out of 233 children (17%). In 13 of these children (33%), the genetic finding was consistent with a diagnosis of GHD. In the remaining 26 children (67%), the analysis revealed an alternative etiology of growth disorder. Specifically, a primary growth plate disorders were found in 8 children (21%), disruption of RAS-MAPK signaling pathway was detected in 13 children (33%), and miscellaneous genetic causes were identified in 5 children (13%). The identified genes associated with these diagnoses are listed in Fig. 1 and Table 2; specific genetic findings are summarized in data provided in an online repository as supplemental materials [12, 20, 23, 28-33] .

**Table 2:** Genetic etiology of a growth disorders stratified by peak stimulated growth hormone concentration

GH_max_, μg/L	GH deficiency	Growth plate disorder	RASopathy	Miscellaneous
	13/39 (33%)	8/39 (21%)	13/39 (33%)	5/39 (13%)
<3.0	*GLI2*	*—*	*NF1*	*PMM2*
	*OTX2* (2)			*LMNA*
	*PROP1*			
	*TBX3*			
	*POU1F1*			
	*GHSR*			
	*KMT2D*			
3.0-4.9	*—*	*COL2A1*	*PTPN11* (2)	*RAI1*
		*NPR2* (3)	*NF1*	*CDC42*
		*FGFR3*		
		*EXT2*		
5.0-6.9	*OTX2*	*FGFR3*	*PTPN11* (2)	*—*
	*SALL4*	*ACAN*	*RAF1*	
	*CHD7*			
	*GNAO1*			
7.0-9.9	*GH1*	*—*	*PTPN11* (3)	*HMGA2*
			*SOS1* (2)	
			*SPRED1*	

Abbreviations: GH, growth hormone; GH_max_, peak stimulated GH concentration.

Analysis of clinical features revealed several differences among the evaluated groups. Specifically, the GHD group exhibited lower maximal stimulated GH concentrations (median 1.8 μg/L [interquartile range (IQR) 1.0-6.3 μg/L] vs 4.8 μg/L [4.0-7.1 μg/L]) compared to the alternative etiology group and 5.9 μg/L (4.1-7.6 μg/L) and the no genetic etiology group (*P* = .017). However, post hoc analysis indicated that the difference between the GHD group and alternative etiology group did not reach statistical significance. Furthermore, the GHD group showed the highest prevalence of combined pituitary hormone deficiency (CPHD; 46% vs 4% and 12%; *P* = .001) and magnetic resonance imaging (MRI) midbrain abnormities (73% vs 14% and 32%; *P* = .003).

Importantly, children in the alternative etiology group demonstrated the least robust response to GH treatment. Despite receiving the highest GH doses, their median height gain after 5 years of therapy was only 1.4 SD (1.0-2.0 SD), compared to 2.3 SD (1.8-3.9 SD) in the GHD group and 1.8 SD (1.4-2.2 SD) in the no genetic etiology group (*P* = .001). Consequently, the alternative etiology group reached the lowest height SDS after 5 years of therapy (−1.8 SD [−2.4 to −1.1 SD] vs −0.8 SD [−2.0 to −0.4 SD] in the GHD group and −1.1 SD [−1.6 to −0.7 SD] in the no genetic etiology group; *P* = .006). A detailed comparison of the groups is provided in Table 3.

**Table 3:** Comparison between children with genetically proven growth hormone deficiency, those with different etiology of short stature elucidated, and those with no genetic etiology found

	GHD group (n = 13)	Alternative etiology group (n = 26)	No genetic etiology group (n = 194)	*P*
Boys	8 (62%)	18 (69%)	135 (70%)	.83
Midparental height (SD)	0.4 (−1.9 to +1.1)	−1.0 (−1.5 to −0.3)	−0.8 (−1.6 to +0.1)	.22
Birth weight adjusted for gestational age (SD)	−0.8 (−1.5 to −0.3)	−0.8 (−1.7 to +0.4)	−0.8 (−1.6 to −0.2)	.84
Birth length adjusted for gestational age (SD)	−1.2 (−1.4 to −0.9)	−1.3 (−2.3 to −0.5)	−1.5 (−2.2 to −0.9)	.32
Neonatal jaundice requiring phototherapy	4 (31%)	7 (27%)	32 (16%)	.52
Neonatal hypoglycemia	2 (15%)	2 (8%)	22 (11%)	.76
Cryptorchidism	2 (25%)	7 (39%)	13 (10%)	.06
Pretreatment IGF-1 below detection limit 15 μg/L	3 (23%)	5 (19%)	17 (9%)	.09
Pretreatment IGF-1 (SD)	−1.8 (−2.0 to −1.6)	−1.6 (−2.1 to −1.2)	−1.6 (−1.9 to −1.3)	.50
Peak stimulated GH, μg/L	1.8 (1.0 to 6.3)*^a^*	4.8 (4.0 to 7.1)*^a,b^*	5.9 (4.1 to 7.6)*^b^*	**.017**
Combined pituitary hormone deficiency	6 (46%)*^a^*	1 (4%)*^b^*	23 (12%)*^b^*	**.001**
MRI performed	11 (85%)	22 (85%)	181 (93%)	.2
MRI midline abnormality	8/11 (73%)*^a^*	3/22 (14%)*^b^*	58/181 (32%)*^b^*	**.003**
Age at GH treatment initiation, y	3.7 (2.3 to 6.7)	4.0 (2.7 to 6.6)	4.8 (3.5 to 6.5)	.24
Height at GH treatment initiation (SD)	−3.4 (−4.4 to −2.8)	−3.0 (−3.3 to −2.8)	−2.9 (−3.2 to −2.6)	.14
GH dose at GH treatment initiation, μg/kg/d	27 (25 to 32)	29 (26 to 32)	28 (25 to 30)	.20
Height after 1 y of GH therapy (SD)	−2.0 (−3.4 to −1.6)	−2.4 (−3.1 to −2.0)	−2.2 (−2.6 to −1.9)	.42
Height gain after 1 y of GH therapy (SD)	0.9 (0.7 to 1.4)	0.7 (0.5 to 0.9)	0.7 (0.5 to 0.9)	.06
GH dose after 1 y of GH therapy, μg/kg/d	25 (23 to 32)	32 (29 to 35)	31 (28 to 33)	.07
Height after 5 y of GH therapy (SD)	−0.8 (−2.0 to −0.4)*^a^*	−1.8 (−2.4 to −1.1)*^b^*	−1.1 (−1.6 to −0.7)*^a^*	**.006**
Height gain after 5 y of GH therapy (SD)	2.3 (1.8 to 3.9)*^a^*	1.4 (1.0 to 2.0)*^b^*	1.8 (1.4 to 2.2)*^a^*	**.001**
GH dose after 5 y of GH therapy, μg/kg/d	31 (28 to 34)*^a^*	35 (32 to 43)*^b^*	32 (29 to 34)*^a^*	**.001**

Continuous variables are expressed as medians (interquartile ranges). *P* values represent the overall difference among groups calculated by Kruskal-Wallis test. Values in bold signify statistical significance.

Abbreviations: GH, growth hormone; GHD, growth hormone deficiency; IGF-1, insulin like growth factor type 1; MRI, magnetic resonance imaging.

*^a,b^* Different superscript letters indicate a statistically significant difference (*P* < .05) between groups based on Dunn's post hoc test with Bonferroni correction for multiple comparisons. Groups sharing a common superscript letter (*^a,b^*) are not statistically different. Categorical variables are expressed as numbers (percentages). *P* values represent the overall difference among groups calculated using the Pearson chi-square test.

*^a,b^* Different superscript letters indicate a statistically significant difference (*P* < .05) based on Fisher exact tests with Bonferroni correction for multiple comparisons.

Among the children enrolled in the study, peak stimulated GH concentrations were distributed as follows: less than 3.0 μg/L (n = 41), 3.0 to 4.9 μg/L (n = 55), 5.0 to 6.9 μg/L (n = 62), and 7.0 to 9.9 μg/L (n = 75). The group with a peak GH less than 3.0 μg/L differed significantly from those with higher peaks across multiple clinical parameters. Specifically, this group presented with higher birth length SD, lower pretreatment IGF-1 concentrations, and a higher prevalence of neonatal hypoglycemia, CPHD, and MRI-detected midline anomalies. Furthermore, these children demonstrated a superior response to GH treatment despite receiving lower GH doses, and their clinical diagnosis of GHD was more frequently confirmed genetically. Importantly, no statistically significant differences regarding clinical parameters, treatment response, or genetic testing results were observed among the different groups with peak GH concentrations greater than 3.0 μg/L. Detailed data are available in Table 4.

**Table 4:** Comparison between children with different peak stimulated growth hormone

GH peak, μg/L	<3.0 (n = 41)	3.0-4.9 (n = 55)	5.0-6.9 (n = 62)	7.0-9.9 (n = 75)	*P*
Midparent height (SD)	−0.1 (−1.2 to +0.5)*^a^*	−1.2 (−1.6 to −0.4)*^b^*	−1.1 (−1.8 to −0.2)*^b^*	−0.8 (−1.5 to −0.2)*^a,b^*	**.02**
Birth weight (SD)	−0.3 (−1.3 to +0.1)	−0.9 (−1.6 to −0.4)	−0.8 (−1.7 to −0.1)	−0.9 (−1.7 to −0.3)	.29
Birth length (SD)	−1.3 (−1.4 to −0.5)*^a^*	−1.7 (−2.4 to −1.1)*^b^*	−1.3 (−2.0 to −0.9)*^a,b^*	−1.6 (−2.2 to −1.1)*^b^*	**.013**
Neonatal jaundice requiring phototherapy	13 (32%)	10 (18%)	9 (15%)	11 (15%)	.18
Neonatal hypoglycemia	13 (32%)*^a,b^*	6 (11%)*^a,b^*	3 (5%)*^b^*	4 (5%)*^b^*	**<.001**
Cryptorchidism	10 (30%)*^a^*	6 (14%)*^a^*	3 (7%)*^a^*	3 (7%)*^a^*	.031
IGF-1 <detection limit 15 μg/L	12 (29%)	4 (7%)	5 (8%)	4 (5%)	**<.001**
Pretreatment IGF-1 (SD)	−1.9 (−2.1 to −1.7)*^a^*	−1.6 (−1.9 to −1.2)*^b^*	−1.6 (−2.0 to −1.3)*^b^*	−1.6 (−2.0 to −1.2)*^b^*	.05
CPHD	18 (44%)*^a^*	5 (10%)*^b^*	3 (5%)*^b^*	4 (5%)*^b^*	**<.001**
MRI midline abnormality	24 (62%)*^a^*	15 (29%)*^b^*	12 (22%)*^b^*	18 (26%)*^b^*	**<.001**
Age at GH treatment initiation, y	4.1 (2.3 to 6.5)	4.2 (3.2 to 6.0)	5.4 (3.7 to 6.7)	4.7 (3.6 to 6.8)	.11
Height at GH treatment initiation (SD)	−3.0 (−3.7 to −2.5)	−3.0 (−3.3 to −2.7)	−2.9 (−3.5 to −2.6)	−2.9 (−3.2 to −2.6)	.83
GH dose at GH treatment initiation, μg/kg/d	26 (24-29)	28 (25-31)	28 (25-32)	28 (26-30)	.17
Height after 1 y of GH therapy (SD)	−2.1 (−2.9 to −1.3)	−2.2 (−2.6 to −1.9)	−2.2 (−2.9 to −2.0)	−2.3 (−2.6 to −2.0)	.26
Height gain after 1 y of GH therapy (SD)	1.0 (0.6 to 1.2)*^a^*	0.8 (0.6 to 1.0*^a,b^*	0.7 (0.5 to 0.9)*^a,b^*	0.7 (0.5 to 0.8)*^b^*	**.011**
GH dose after 1 y of GH therapy, μg/kg/d	27 (23-31)*^a^*	31 (27-33)*^b^*	31 (29-34)*^b^*	31 (29-34)*^b^*	**<.001**
Height after 5 y of GH therapy (SD)	−0.8 (−1.8 to 0.0)*^a^*	−1.2 (−1.6 to −0.6)*^a,b^*	−1.3 (−2.0 to −0.8)*^b^*	−1.1 (−1.8 to −0.9)*^a,b^*	**.024**
Height gain after 5 y of GH therapy (SD)	2.1 (1.6 to 3.0)*^a^*	2.0 (1.4 to 2.3)*^a,b^*	1.7 (1.4 to 2.2)*^a,b^*	1.7 (1.2 to 2.0)*^b^*	**.012**
GH dose after 5 y of GH therapy, μg/kg/d	30 (24-33)*^a^*	32 (30-34)*^a,b^*	33 (30-35)*^b^*	33 (30-35)*^b^*	**.002**
Genetic etiology elucidated	11(27%)	11 (20%)	9 (15%)	8 (11%)	.13
Genetically confirmed GHD	8 (20%)*^a^*	0 (0%)*^b^*	4 (6%)*^a,b^*	1 (1%)*^b^*	**<**.**001**

Continuous variables are expressed as medians (interquartile ranges). *P* values represent the overall difference among groups calculated by Kruskal-Wallis test. Values in bold signify statistical significance.

Abbreviations: CPHD, combined pituitary hormone deficiency; GH, growth hormone; IGF-1, insulin like growth factor type 1; MRI, magnetic resonance imaging.

*^a,b^* Different superscript letters indicate a statistically significant difference (*P* < .05) between groups based on Dunn's post-hoc test with Bonferroni correction for multiple comparisons. Groups sharing a common superscript letter (*^a,b^*) are not statistically different. Categorical variables are expressed as numbers (percentages). *P* values represent the overall difference among groups calculated using the Pearson chi-square test. *^a,b^*Different superscript letters indicate a statistically significant difference (*P* < .05) based on Fisher exact tests with Bonferroni correction for multiple comparisons.

## Discussion

In this study, we comprehensively evaluated the genetic etiology of short stature in a relatively large cohort of children initially diagnosed with GHD. Our findings reveal substantial genetic heterogeneity, with a statistically significant proportion of identified causative variants involving mechanisms not directly related to GH production or secretion. Furthermore, we identified distinct differences in clinical phenotypes and responses to GH treatment based on the results of genetic testing.

Modern genetic methods, particularly NGS, have repeatedly demonstrated their value in identifying the pathogenesis of growth disorders. Across multiple cohorts, these methods have clarified the etiology of short stature in 10% to 44% of children [12, 13, 16, 20, 34-36]. In line with these observations, our study advances the understanding of the etiology of short stature in children clinically diagnosed with GHD. Unlike previous studies that focused primarily on genes directly associated with GHD [18, 37-39], we systematically assessed both traditional GHD genes and genes implicated in alternative mechanisms of impaired growth. This comprehensive strategy offers a unique perspective on this cohort.

In our study population, causative variants in genes directly involved in GH secretion accounted for 33% of cases with an identified genetic etiology. This group comprised genes well established in the pathogenesis of GHD (*GH1*, *GHSR*, *PROP1*, *POU1F1*, *GLI2*, *OTX2*, *TBX3*, *CHD7*, and *KMT2D*) [10, 37, 40-47], as well as 2 additional genes likely involved in the regulation of GH secretion. The *SALL4* gene encodes a transcription factor with a unique role in early embryonic development. It is expressed in embryonic pituitary and midbrain structures, where it acts as a crucial cofactor for the Sonic Hedgehog signaling pathway and the *SOX2* transcription factor, both of which are essential for the proper development of the hypothalamic-pituitary axis, as we previously reviewed in more detail [33, 48]. Similarly, the *GNAO1* gene is crucial for the regulation of neuronal development [49]. In children with pathogenic variants in this gene, impaired development of corpus callosum is a common feature [50], highlighting the importance of this gene in the development of the midbrain.

In the remaining 67% of children with an identified genetic etiology, causative variants were found in genes not traditionally associated with GHD. Notably, a substantial proportion of these children (13/39; 33%) were diagnosed with RASopathies. The pathophysiology of growth impairment in these conditions is complex and likely multifactorial [51]. Although GHD has been reported in these patients [52, 53], it is not considered the primary driver of growth failure. Instead, the current literature suggests that hyperactivation of the RAS-MAPK pathway leads to GH insensitivity and directly compromises growth plate function [51, 54, 55]. In addition, a substantial number of children (8/39; 21%) had a primary growth plate disorder. Even among the 5 children with miscellaneous genetic etiologies, GHD does not appear to be the primary pathogenic mechanism. For instance, congenital disorders of glycosylation caused by *PMM2* gene variants impair growth by affecting the stability and function of IGF-1 signaling components [56, 57]. The *LMNA* gene encodes lamin A, a key protein of the nuclear lamina, whose alteration disrupts the integrity of the inner nuclear membrane [58]. Similarly, pathogenic variants in *HMGA2* and *CDC42* genes affect critical cellular functions, including cell cycle regulations [59, 60]. Finally, Smith-Magenis syndrome represents a complex disorder in which short stature is frequently associated with typical signs of bone dysplasia such as scoliosis or brachydactyly [61].

The frequent discrepancy between clinical diagnosis of GHD and the identified genetic etiology of short stature may have several explanations. First, the reliability of methods currently used to diagnose GHD has been repeatedly questioned [4, 9, 62]. Given the low estimated prevalence of GHD and the high rate of false-positive results associated with GH stimulation testing, Bright et al [63] calculated the probability of a true-positive test in a child with short stature to be less than 3%. Furthermore, Dauber et al [10] hypothesized that children with diagnosed GHD represent a heterogeneous group with etiologies often independent of GH secretion, a hypothesis strongly supported by the results of our study. On the other hand, we must acknowledge the potential for false-positive genetic results, particularly when variants classified as “likely pathogenic” are considered causative. However, since the probability of pathogenicity for likely pathogenic variants is greater than 90% [26], this diagnostic evaluation offers superior reliability compared to traditional GHD testing methods. It is plausible that in some individuals, GHD and alternative genetic etiology may coexist, contributing synergistically to the growth disorder.

Notably, the 3 evaluated groups (GHD group, alternative etiology group, and no genetic etiology group) differed substantially in their clinical presentation. Consistently with general assumptions, children with genetically confirmed GHD presented more frequently with CPHD and MRI midbrain abnormalities. Moreover, the GHD group exhibited the lowest peak stimulated GH concentrations, with 8 out of 13 patients meeting the criteria for severe GHD (peak GH <3 μg/L). Conversely, one proband carrying pathogenic variant p.Arg209His in the *GH1* gene demonstrated a relatively high stimulated GH concentration (8.1 μg/L), a value that according to some guidelines would exclude GHD [1,6]. This observation is consistent with a report from Argentina, where carriers of this specific variant also presented with relatively high stimulated GH [64]. Functional studies explain this discrepancy. This variant does not abolish GH bioactivity but rather impairs the regulation of its secretion. The altered GH molecule is retained intracellularly and secreted in decreased amounts or with a delay [65], a defect that might be masked in the nonphysiological conditions of stimulation testing.

Despite the global use of GH stimulation tests to confirm GHD, diagnostic cutoffs remain arbitrary, ranging from 6.7 to 10.0 μg/L depending on the country [6]. Our findings highlight distinct phenotypic profiles and significantly better treatment outcomes in children with peak GH levels < 3.0 μg/L, aligning with the criteria for severe GHD. In contrast, the various groups with a peak GH > 3.0 μg/L exhibited no significant phenotypic or therapeutic differences. Adopting the stricter 7.0 μg/L threshold recommended by recent guidelines [1] would substantially (by 32% in our study cohort) reduce the number of children diagnosed with GHD. However, given the lack of distinction between these children and those with peak stimulated GH concentration of 3.0 to 6.9 μg/L, our data suggest that this adjustment would not optimize diagnostic accuracy or GH treatment efficacy.

Given that idiopathic short stature is not currently an approved indication for GH treatment in Europe, the diagnosis of GHD carries considerable importance, often serving as the sole access to the therapy. Importantly, a presumed false-positive GHD diagnosis does not preclude a therapeutic benefit. Although children with an identified alternative genetic etiology exhibited a less robust response to GH therapy despite receiving higher GH doses, they achieved substantial growth improvement (a median of 1.4 SD after 5 years). Consequently, refining the current indication criteria for GH therapy represents a major challenge for contemporary pediatric endocrinology. We suggest that until comprehensive data regarding the efficacy of GH in specific genetic disorders become available, the debate regarding the approval of idiopathic short stature as an indication in Europe warrants reinitiation.

Recent international guidelines for the genetic evaluation of short stature [66] recommend a 2-tier approach for children with severe GHD: an initial targeted panel for GHD-causing genes, followed by an expanded evaluation if first-line testing is negative. Our data support this strategy, as 70% (7/10) of the children in our cohort with a peak GH < 3.0 μg/L and an identified genetic etiology harbored variants directly associated with GHD. Although current guidelines do not recommend routine genetic testing in children with a peak GH > 3.0 μg/L in the absence of additional phenotypic features, our findings indicate that a genetic etiology of short stature can still be identified in a considerable proportion of this group at a rate comparable to that of children with isolated short stature [67]. Given the predominance of variants in genes not affecting GH secretion in this cohort, a comprehensive approach encompassing a broader array of short stature genes should be implemented from the outset.

Our study has several strengths. Most important, the relatively large cohort size, combined with a high enrollment rate, mitigates the risk of potential selection bias and enhances the robustness of our findings. However, we must acknowledge several limitations. First, functional validations of identified novel variants were not performed. Second, noncoding variants (except for intron-exon boundaries) were not captured by NGS. Finally, although our NGS panel covered a comprehensive set of 398 growth disorder–associated genes, causative variants in genes not included in this panel could have remained undetected. Detailed anthropometric measurements, including the evaluation of body proportionality or facial recognition software that could potentially help optimize phenotypic evaluation and candidate gene selection, were not used. Finally, although a comprehensive clinical evaluation was regularly performed in all patients, we recognize that IGF-1 levels can be influenced by non–GH-dependent factors such as nutritional status or illness.

To conclude, the clinical diagnosis of GHD frequently masks a wide spectrum of underlying genetic diagnoses, ranging from typical GHD genes to primary growth plate disorders and disruption of fundamental intracellular processes, particularly the RAS-MAPK signaling pathway. Our results highlight the limitations of current GH stimulation tests and support the integration of broad NGS panels into the genetic investigation of children diagnosed with GHD. To improve the cost-effectiveness and accessibility of genetic testing, further studies are needed to identify specific clinical features predictive of a monogenic etiology in children with diagnosed GHD.

## Data Availability

Some or all datasets generated during and/or analyzed during the current study are not publicly available but are available from the corresponding author on reasonable request.

## Abbreviations

CPHD: combined pituitary hormone deficiency
GH: growth hormone
GHD: growth hormone deficiency
IGF-1: insulin-like growth factor type 1
IQR: interquartile range
MRI: magnetic resonance imaging
NGS: next-generation sequencing
